# Towards a More Reliable Identification of Isomeric Metabolites Using Pattern Guided Retention Validation

**DOI:** 10.3390/metabo10110457

**Published:** 2020-11-12

**Authors:** Tobias Opialla, Stefan Kempa, Matthias Pietzke

**Affiliations:** Integrative Metabolomics and Proteomics, Berlin Institute of Medical Systems Biology/Max-Delbrück Center for Molecular Medicine, 13125 Berlin, Germany; tobias.opialla@mdc-berlin.de

**Keywords:** metabolomics, LC–MS, GC–MS, chromatography, retention index, identification, standardization

## Abstract

Reliable analyte identification is critical in metabolomics experiments to ensure proper interpretation of data. Due to chemical similarity of metabolites (as isobars and isomers) identification by mass spectrometry or chromatography alone can be difficult. Here we show that isomeric compounds are quite common in the metabolic space as given in common metabolite databases. Further, we show that retention information can shift dramatically between different experiments decreasing the value of external or even in-house compound databases. As a consequence the retention information in compound databases should be updated regularly, to allow a reliable identification. To do so we present a feasible and budget conscious method to guarantee updates of retention information on a regular basis using well designed compound mixtures. For this we combine compounds in “Ident-Mixes”, showing a way to distinctly identify chemically similar compounds through combinatorics and principle of exclusion. We illustrate the feasibility of this approach by comparing Gas chromatography (GC)–columns with identical properties from three different vendors and by creating a compound database from measuring these mixtures by Liquid chromatography–mass spectrometry (LC–MS). The results show the high influence of used materials on retention behavior and the ability of our approach to generate high quality identifications in a short time.

## 1. Introduction

Mass-spectrometry (MS) coupled to chromatography is a widely used approach for metabolomics analyses. Typical setups include gas chromatography mass-spectrometry (GC–MS), liquid chromatography mass-spectrometry (LC–MS) and capillary electrophoresis mass-spectrometry (CE–MS). In these setups both chromatography and mass-spectrometry cooperate to the identification of metabolites, fulfilling the request for two orthogonal measures needed for a reliable identification [1,2]. Coeluting metabolites can often be separated by specific masses, while metabolites with similar MS-properties may be separated by chromatography. There are some basic differences between GC–MS and LC–MS (Table 1). GC–MS typically offers higher chromatographic resolution, peak widths are smaller, and a retention index (RI) system is often used [3]. This renders chromatographic identification more robust than relying solely on retention time. On the other hand, coupled mass-spectrometers are often low-resolution (time of flight or triple-quadrupol detectors), although high-resolution GC–MS does exist. LC–MS typically includes high resolution mass spectrometry (e.g., Orbitrap detectors), but in LC peaks are often wider, and coelution occurs more frequently. Additionally, despite some efforts [4,5,6,7,8,9] retention index or relative retention times are not routinely used, so the identification is dependent on the retention time, which often shows much higher fluctuations as the retention index.

Nevertheless, in both setups reliable separation of isomeric metabolites is difficult. This includes some prominent examples for metabolites in the central carbon metabolism as citric acid and isocitric acid, leucine and isoleucine (as well as norleucine, aminocaproic acid and β-leucine) and hexoses as glucose, fructose and galactose (but also mannose, inositol, sorbose and others). The analytical challenge is to separate and identify very similar (as isomeric and isobaric) metabolites, often fulfilling different biological functions. Moreover, the analyst should know the abilities but also ambiguities of systems to identify and quantify the difficult separation cases.

To address this problem, we first investigated the occurrence of isomeric metabolites in widely used metabolite databases (HMDB and KEGG). We show examples how compound derivatisation can help to separate some cases of isomerism and that sole reliance on retention database information can be difficult. As a consequence, validating and updating the retention behaviour using the exact same setup is required to warrant identification [1,2]. To improve identification of isobaric and isomeric metabolites we have introduced a strategy to verify the retention behaviour for a high number of metabolites simultaneously. This leads to higher identification confidence, simplifies and accelerates the data processing.

## 2. Results

### 2.1. The Majority of Metabolites Are Isobars

The chemical space observed in metabolomics is complex due to the combination and permutation of common side groups along carbon backbones with different lengths and shapes. In order to define how dense the biologically relevant chemical space is, we explored sum formulae and compound masses stored in the human metabolome database (HMDB) [10] and the Kyoto Encyclopedia of Genes and Genomes (KEGG) [11].

From the KEGG database we extracted 7133 metabolites with a defined sum formula and an associated enzymatic reaction (KEGG-all). Overall these metabolites have 3877 unique masses. Focussing on human metabolism we excluded plant secondary metabolites and bacteria specific metabolites, and consequently identified 2504 metabolites in pathways being present in humans (“KEGG-human”), with 1589 unique masses (Table S1), so each mass represents 1.6 metabolites on average.

**Figure 1:**
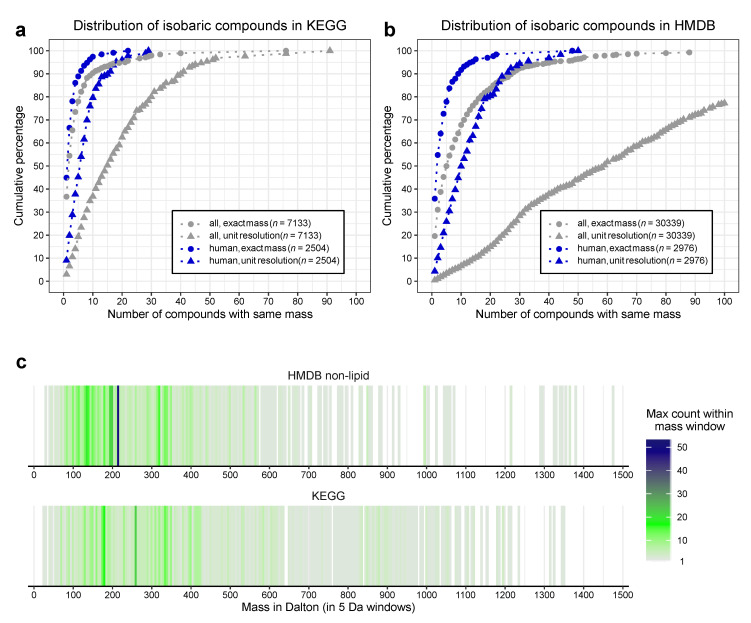
Compound similarities in common metabolite databases. (**a**) Cumulative percentage of isobaric metabolites extracted from the KEGG-database. “All” denotes organic metabolites with associated enzymatic reaction, “human” a subset with associated pathways in humans, “exact masses” indicate the exact mass as contained in the database, while “unit resolution” counts the frequencies with rounded masses. (**b**) Cumulative proportion of isobaric metabolites extracted from the human metabolome database (HMDB). “All” denotes non-lipid metabolites in the HMDB, while “human” denotes metabolites listed as “detected” or “quantified”. (**c**) Distribution of shared masses over the mass-range. Isomers were counted on the respective human dataset for exact mass. Each line represents a 5 Da mass window, with the maximum of the counts within the window indicated by colour.

As shown in Figure 1a (and Table S2) even for the exact masses (circles) only a minority of metabolites (37% for KEGG-all, 45% for KEGG-human) have unique sum-formulae and molar masses. The majority of metabolites share their mass with at least one additional compound. One fifth of the metabolites have at least three (KEGG-human) or five (KEGG-all) isobaric metabolites in the database. As expected, this effect is more pronounced for unit-resolution mass values (triangles). Less than 10% of the metabolites have unique nominal masses and 50% of the metabolites in the KEGG-human dataset have more than five isobars. KEGG-all contains at least 15 isobars for 50% of the metabolites (Figure 1a).

Similar results are found in the HMDB. We extracted the information of 109,573 metabolites. The HMDB (in contrast to the KEGG) contains more lipids (79,224 of these 109,573 metabolites). Lipids are known to have a high isobaric count, due to the combinatorial space of different fatty acids with the head-group. This likely influences the interpretation, so we decided to focus on the non-lipid species, removing the lipids from the analysis. From the remaining 30,339 metabolites (HMDB-all) we created a “HMDB-human” subset based on their state as “detected” or “quantified” (2976 metabolites, Table S1). This number of substances is comparable to the KEGG-human subset, showing the congruence of both approaches.

In line with this, the cumulative distributions are very similar, although the isomer-count is much higher in the HMDB (Figure 1b and Table S3) Here only 19.6% (in HMDB-all) or 35.8% (in HMDB-human) of the exact masses are exclusive to a single compound. This decreases for unit resolution to 0.5% (HMDB-all) and 4.33% (HMDB-human).

The number of isobars distributes over the whole mass range in both human datasets (Figure 1c). Even though the pure chemical complexity is expected to increase with higher masses, the biological relevant complexity is highest for relatively small compounds with a mass of 100–400 Da. This supports the “building block” concept in biology as very large compounds are typically made of combining a limited set of smaller units.

The metabolites with the highest isomer count in KEGG-all are hexose-phosphates ($C_{6}H_{13}O_{9}P$ with 22 isomers), hexoses ($C_{6}H_{12}O_{6}$ with 17 isomers), a hexose-lactone ($C_{6}H_{10}O_{6}$ with 13 isomers) and a leukotriene B4 ($C_{20}H_{32}O_{4}$ with 13 isomers) (Table S4). Even after removing lipids, the HMDB dataset still contains apolar metabolites among the metabolites with the highest isomer-count (Table S5). This is exemplified by hydroxyoct-enoylglycine ($C_{10}H_{17}{\mathrm{NO}}_{4}$, 215.25 Da with 48 isomers), β-selinene (or α-bergamotene, $C_{15}H_{24}$, 204.35 Da with 22 isomers), octadienoylglycine ($C_{10}H_{15}{\mathrm{NO}}_{3}$, 197.23 Da with 21 isomers), d-limonene ($C_{10}H_{16}$, 136.23 Da with 19 isomers), caprylic acid ($C_{8}H_{16}O_{2}$, 144.21 Da with 15 isomers) and an epoxy-eicosatrienoic acid ($C_{20}H_{32}O_{3}$, 320.47 Da with 15 isomers). The hexoses follow with 14 isomers, leukotriene B4 is listed with 12 isomers, the hexose-phosphates with seven isomers and hexose-lactones with just two isomers, illustrating some of the differences between the two databases.

Many of the similarities include small molecular weight metabolites as found in the central carbon metabolism and not only complex secondary metabolites. Metabolites with identical sum-formula and mass often fulfil different roles in metabolism, depending on the type of the functional groups (lactate vs. glyceraldehyde) the position of functional groups (citrate vs. isocitrate, leucine vs. isoleucine), the presence of primary, secondary or tertiary amines (alanine vs. sarcosine), the cis- or trans-orientation of double bonds (fumarate vs. maleate) and even the orientation of functional groups along the backbone (fructose vs. glucose). This illustrates that analytical separation and unambiguous identification (and ultimately quantification) of metabolites that differ only in the localisation and orientation of functional groups is an important analytical challenge.

### 2.2. Derivatisation and Fragmentation Add Discriminatory Elements to Separate Isomeric Metabolites

**Figure 2:**
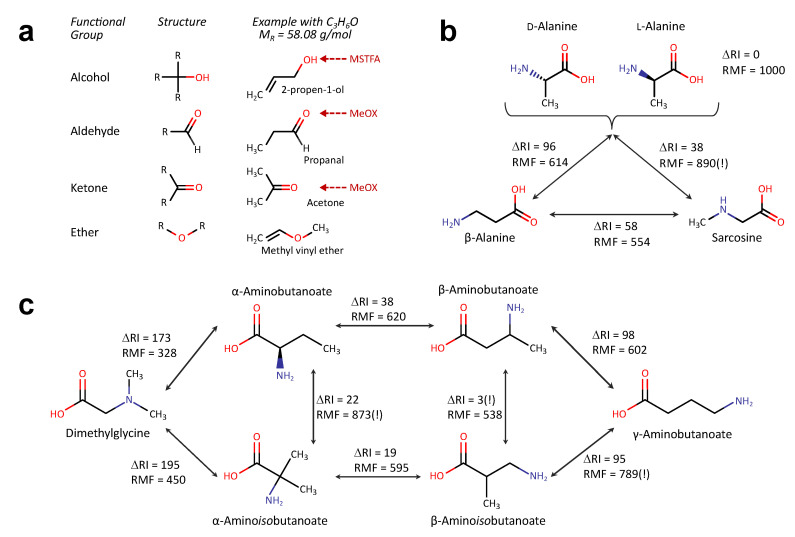
Discrimination of different functional groups by derivatisation. (**a**) Scheme illustrating the accessibility of different functional groups towards silylation (MSTFA) and methoxymation (MeOX). (**b**,**c**) Pairwise comparison of retention indices (RI) and reverse match factor (RMF) for metabolites with sum formula $C_{3}H_{7}{\mathrm{NO}}_{2}$ (detected as 2-TMS product) (**b**) and metabolites with sum formula $C_{4}H_{9}{\mathrm{NO}}_{2}$ (**c**). ΔRI: difference in retention index, stored in the GMD for metabolites with comparable derivatisation states (similar RI window), RMF: similarity of mass spectra obtained in pairwise comparison (0: no similarity—1000: equality).

Derivatisation, a prerequisite for GC–MS based metabolomics, masks hydrophilic functional groups to increase volatility of the metabolites enabling a separation in the gas-phase [12]. Additionally, this can help identifying metabolites as different functional groups may be differentially accessible to the derivatisation reactions. For example, in a widely used protocol, first aldehyde and ketone groups undergo methoximation, followed by silylation of alcohols and amines through MSTFA-treatment [12]. As this requires polar N-H, O-H or S-H bonds, tertiary amines or ether-bonds are not modified at all (Figure 2a). As a consequence, structural isomers can generate different derivatisation products, differing in their retention behaviour, molecular masses and fragments.

To illustrate this, we tested the similarities of some structural isomers after derivatisation and GC–MS detection at the levels of retention time and mass-spectra similarity. For this we used the information available in the Golm Metabolome Database (GMD) [13] and compared the mass-spectra similarities using MSSearch 2.0 (AMDIS). This gives the reverse match factor (RMF) [14], where 0 indicates no similarity and 1000 a perfect match.

First we tested metabolites similar to alanine (Figure 2b), with a sum formula of $C_{3}H_{7}{\mathrm{NO}}_{2}$ and a mass of 89.0477 g/mol. The HMDB lists five metabolites with this formula: β-alanine, d-alanine, l-alanine, ethyl carbamate and sarcosine. The KEGG additionally lists 2-nitropropane and N-hydroxymethyl-N-methylformamide. Of these metabolites the GMD includes dl-alanine, β-alanine and sarcosine. The stereo-isomers of alanine can only be separated with stereospecific columns [15]. Alanine and β-alanine or β-alanine and sarcosine can easily be separated from each other by both retention index and spectra similarity The comparison between sarcosine and alanine delivers a similarity score of 890 (Figure 2b), this could lead to a false identification. Fortunately, the retention indices differ sufficiently to prevent this (ΔRI = 38).

As an example for a another versatile sum formula, we then compared metabolites with the sum formula $C_{4}H_{9}{\mathrm{NO}}_{2}$ (Figure 2c). In total, 12 and 14 different molecules are reported in the KEGG or HMDB databases, respectively; the GMD lists six of these metabolites. The majority of these metabolites differ by amino group position (α-, β-, or γ-position) or the structure of the carbon-backbone (butanoic acid versus isobutanoic acid). While most of these metabolites are reported to be present in human tissues or body fluids, only few of them are known to fulfil biological functions, e.g., γ-aminobutanoate (GABA) as neurotransmitter, β-aminoisobutanoate as catabolic product of pyrimidines and valine or with dimethylglycine as intermediary step in choline to glycine conversion. Chemically dimethylglycine is the most distinct metabolite, as this can only bind one TMS group, consequently it differs strongly from the other metabolites with respect to RI and RMF. α-aminobutanoate and α-aminoisobutanoate have similar mass-spectra whereas β-aminobutanoate and β-aminoisobutanoate have a very similar retention (but different spectra). There is also a spectra-similarity higher than 700 between β-aminoisobutanoate and γ-aminobutanoate. In summary, all of the metabolites can be differentiated either by retention or spectral differences.

### 2.3. Enantiomers Can Be Separated by Chromatographic Means

**Figure 3:**
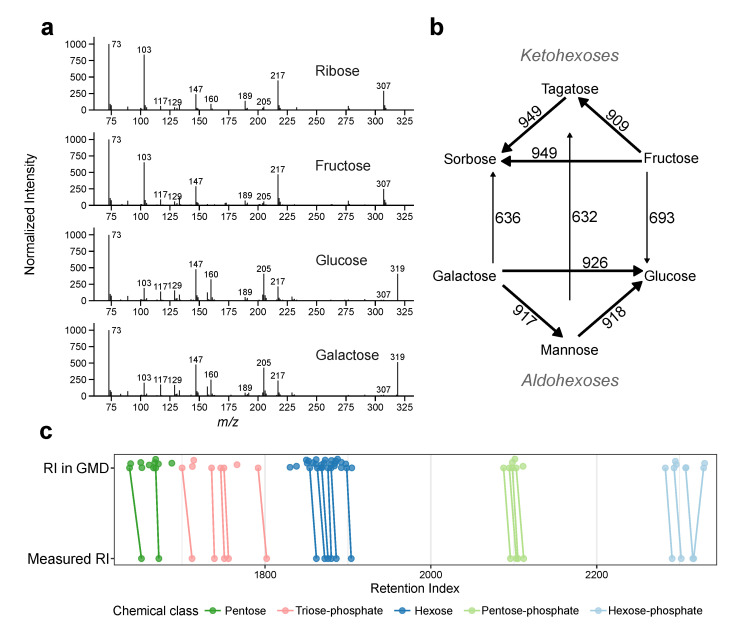
Mass spectra- and retention time similarities between sugars in measured by GC–MS. (**a**) Electron impact mass spectra of ribose (an aldopentose), fructose (a ketohexose), glucose and galactose (aldohexoses), note their similarity. (**b**) RMF-values between GMD spectra; ketohexoses (top) and aldohexoses (bottom). Arrows indicate direction of comparison (**c**) Retention indices as stored in the GMD (top) and measured on our system (bottom). Colours indicate groups of sugars, thus inherently similar compounds. Compounds detected in both setups are connected by lines to show the shifts between the systems. Note, that the shifts are not always identical and span over several other compounds in the GMD with similar mass spectra.

Next, we examined how well enantiomers can be separated by GC–MS. For this, we analysed the mass-spectra similarities (RMF) and retention time differences (ΔRI) of compound information stored in the Golm Metabolome Database (GMD) [13]. We focussed on sugars and sugar-phosphates, as these metabolites have a high number of isomers and are well represented in the GMD. Due to the high structural similarity they also show high RMF (Figure 3a). Even ribose (a pentose) has a fragmentation pattern similar to fructose (a hexose). This is reflected in RMF in between the three ketohexoses (fructose, tagatose and sorbose) and in between the three aldohexoses (glucose, mannose and galactose) with RMF > 900 (Figure 3b). Nevertheless, the group of aldohexoses and the group of ketohexoses remain distinguishable with similarity scores of 600–700.

Additionally, we compared the retention indices (RIs) between sugars or sugar-phosphates stored in the GMD (Figure 3c, top) with RIs measured on our system (Figure 3c, bottom). Compounds included in both setups show retention index shifts (ΔRI), that are not necessarily parallel. The average ΔRI between the two setups is 7.6 RI units. Within shift range, there are multiple entries listed in the GMD, especially for compound classes with multiple similar metabolites, as hexoses. It is worth to note that we use a very similar setup (column-type, temperature gradient, retention index system) as the one used to create the GMD (see Section 4.5.2 methods and Table S10). As the retention index is more robust, than the retention time, we did not expect big ΔRI-values (Table S6). This illustrates how an identification using only library data can be ambiguous, as RMF-values are high. As a conclusion authentic standards should be tested using exactly the same setup to validate the retention information.

### 2.4. The Retention of Multiple Metabolites Can Be Simultaneously Validated with Appropriately Designed Mixtures

To reliably identify metabolites, it is therefore essential to measure authentic standards with the exact same setup as used for the samples. However, as this is time- and labour intensive this is often done only once, storing the retention information in a custom database. Unfortunately, chromatographic columns will age over time and even identical machines can perform differently. This renders identification of metabolites less reliable over time. We observed the necessity to adjust retention times in LC–MS by 20–30 s between experiments run on different dates (not shown), which is also in the range of retention time differences between similar metabolites. Furthermore, in GC–MS we observed considerable RI-shifts between column vendors even with the same coating materials. More confidence can be reached when the retention of authentic standards is checked more frequently e.g., monthly or weekly as part of the quality-control routine.

**Figure 4:**
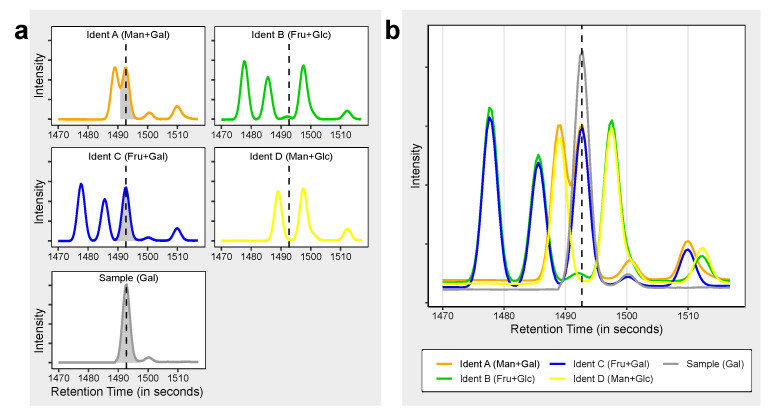
Application of the Ident-Mix for compound identification. (**a**,**b**) Illustration of retention validation using Ident-Mixes. four hexoses (fructose, glucose, galactose, mannose) with similar retention present in four different mixtures (Ident A to Ident D) and compared to a sample containing galactose. (**a**) shown with separate window for each compound as seen e.g., with Thermo Fisher Tracefinder software (**b**) Shown in overlay mode as seen e.g., with LECO ChromaTOF software.

In order to save measurement time as many metabolites as possible should be validated at once. However, if dozens or hundreds of metabolites are tested simultaneously the same challenge of potentially unclear identifications arises again. We solved this issue by distributing the metabolites over four different mixtures (A, B, C, D) in a way that the same compound appears in two of these mixtures, with similar metabolites present in a different combination of these mixtures (Table 2 and Figure 4a,b). We term these mixtures “Ident-Mixes” as they are used to facilitate compound identification. This approach decreases the complexity in each mixture by 50% and at the same time adds some redundancy. Due to the different possible combinations, this allows to differentiate up to six similar metabolites (present in Ident-Mix A&B, A&C, A&D, B&C, B&D, C&D). Additionally, pairs of potentially coeluting compounds can be distributed fully separated (Mixes B&C vs. Mixes A&D, see Fructose/Mannose in Table 2) or in a way that allows to monitor if they can still be separated when simultaneously present (Mixes A&D vs. Mixes A&C, see Mannose/Galactose). More flexibility and capacity can be reached by using five mixes (10 possibilities) or six mixes (15 possibilities). With this approach we routinely validate the retention behaviour of 105 metabolites measured by GC–MS with just four additional measurements in each sample batch. These mixtures cover the a wide range of the metabolic pathways in humans (Supplementary Figure S1), their composition is reported in Table S7.

To use this information we typically match the measurements of the Ident-Mix against the Ident-Mix spectra databases. We identify the compounds based on their mass spectra similarity, retention behaviour and their combinatorial pattern. We update the retention information of these compounds and consequently identify the measured samples in the same batch against this updated database.

### 2.5. With the Help of These Mixtures It Possible to Transfer Compound Identifications between Different Setups

**Figure 5:**
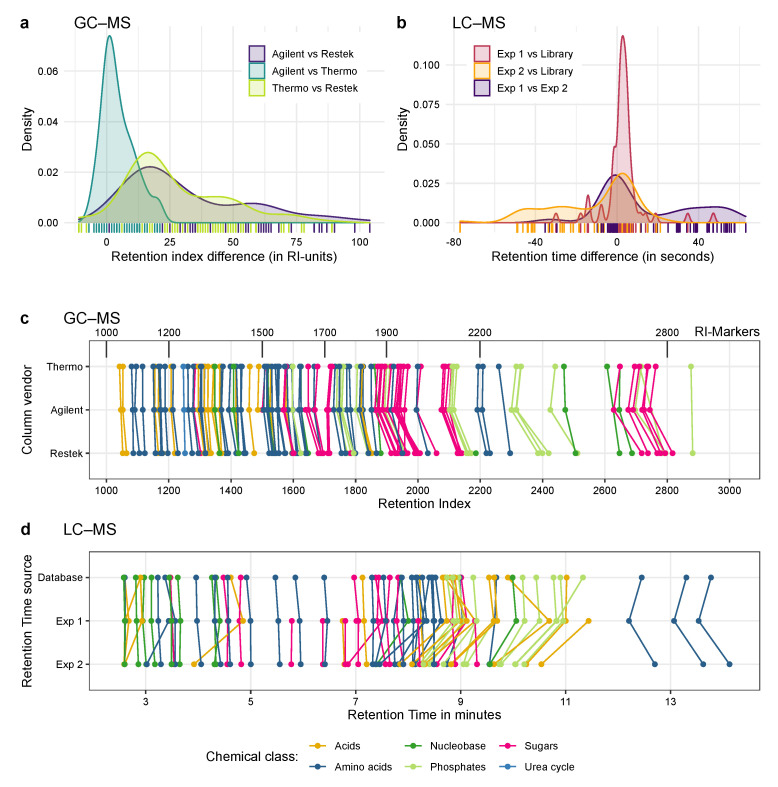
Application of the Ident-Mix for retention validation and compound identification by GC–MS and LC–MS. (**a**,**c**) Testing retention of metabolites by GC–MS with three GC columns (same polarity, different vendors) and identical machine and temperature program. (**b**,**d**) Testing retention of metabolites by LC–MS with two different LC columns (same make and model) and identical machine solvent gradient. (**a**) Histogram showing the RI differences for the GC-experiment, overall the columns from Agilent and Thermo are the most similar. (**b**) Histogram showing the rt-differences for the LC-experiment, here experiment 1 matches the database entries more closely than the second experiment. (**c**) Retention indices of compounds in the GC-experiment for the three setups. Coloured lines connect the same metabolite, black lines denote RI-markers. If a point is missing, no peak was detectable. (**d**) Retention times of compounds in the LC experiment for the database and two measurements. Coloured lines connect the same metabolite, if a point is missing no peak was detectable or the metabolite was not present in the in-house database. Overall RT values from experiment 1 are closer to the library entries than experiment 2.

We further compared the retention behaviour of three different GC–MS columns with the same polarity and nominal coating material but from different vendors. Figure 5a shows substantial retention index shifts between the different vendors. More surprisingly the amount of the RI shift differs not even between metabolites of different classes but even within the same compound class (Figure 5c, and Table S8). This leads to changes in the elution order of some compounds, rendering identification based solely on database information more complicated. Using the Ident-Mixes it was possible to identify the compounds reliably, demonstrating the benefits of this approach.

We additionally measured the Ident-Mixes by high-resolution LC–MS to further test whether these mixtures can be used to transfer compound identifications between different systems. The obtained masses lead to predicted sum formulae and suggested compound identifications. These putative identifications were compared against the given Ident-Mix pattern. The most intense peak matching the expected pattern was used as identification. From these identifications, we created a compound library based on *m/z* and retention time. The names in the library contained the name of the metabolite, its Ident-Mix pattern and the measured retention time for an easy comparison (see discussion). This library was created within one working day and was then matched against two different measurements, performed 11 months apart. This matching was finished within a few hours.

The majority of the compounds contained in the mixes, which were initially optimized for GC–MS measurements, could also be detected and separated by the used LC–MS method, demonstrating the separation power of the SeQuant ZIC-pHILIC column. Some of the compounds could not be separated completely; here a manual processing with smaller allowed retention time windows could help, albeit this also increases the risk of false negative results (compounds present but shifted outside the allowed windows).

To validate the results, we compared the obtained retention times against the information stored in a in-house database generated by measuring individual compounds with this setup. As shown in Figure 5b in at least the first experiment the measured retention time matches quite closely to the RT stored in the database, with the mean of the retention time difference of just 5.4 s. This indicates that the identifications are likely to be correct. 11 compounds could be identified that were not yet included in the database and for one compound we found the stored retention time to be wrong. Nevertheless, there are a few compounds with retention time variations of up to 50 s. More surprisingly, there is a higher variation between the two different measurements compared to the database entries. In the second experiment the majority of metabolites have a RT difference (between the two experiments and the experiment and the database) ranging from −20 and +20 s. Additionally there is a high fraction of metabolites with a RT difference around 40 to 50 s. As shown in Figure 5d (and Table S9) the retention time shifts also differ between the metabolites and their classes. While the sugars and early amino acids elute quite consistently, most of the acids, phosphates and nucleobases are shifted to earlier time points, and the late eluting amino acids are shifted to later time points between the two experiments.

## 3. Discussion

As illustrated in Figure 1 the majority of human metabolites share their molecular mass and sum formula with at least one additional metabolite, so they cannot be identified unambiguously by their mass alone, not even with high-resolution mass-spectrometry. As a consequence, an orthogonal approach is essential to truly identify metabolites in MS-based experiments, using at least one additional orthogonal readout, e.g., the retention behaviour. Additionally, ion mobility can be employed to better separate isomeric compounds; however, this technique is less common [16]. When a definite mass-spectrometric identification isn’t possible, chromatographic separation becomes the limiting factor. This is even more important for mass-spectrometers with low or unit resolution. Every compound that is included in routine identification should be inspected if possible isobars can lead to misidentifications. Further potential ambiguities may occur due to in-source fragmentation (e.g., from malate to fumarate) [17] or derivatisation products that can be generated from different educts (e.g., pyroglutamate from both glutamate and glutamine) [18]. Consequently, experience is needed to reliably identify metabolites.

To address this issue, we present a strategy that enables the validation of retention information for multiple compounds simultaneously. The measurement of the Ident-Mixes extends the measurement time and machine occupancy only slightly, but increases confidence in the results. The researcher immediately knows which metabolites can or cannot be separated with the used system. In the latter case bottlenecks are identified that might need method improvement. Every compound being detected in the Ident-Mixes and not found in the samples can be seen as “detectable but not found”. This can be due to true negative readings (metabolites that are absent or present with a concentration below detection limit), or it can be a result of signal suppression or overlapping peaks from the sample matrix. This procedure simplifies the analysis and renders it less dependent on expert knowledge. We observed that starters in the field were able to deliver high-quality identifications in similar time-frame as experts working in the field for several years. Further it is advantageous compared to updating retention information using biological samples, especially for conditions in which metabolites are not always detected e.g., due to varying sample matrices. As a consequence, the obtained identifications are of high quality. This is particularly helpful when data is shared with other labs or stored in digital repositories [19]. By reporting the composition of the used Ident-Mixes and the measured retention times other users can reproduce the validity of the identification and its limits even without checking the raw-data.

We developed this strategy for GC–MS based analysis of mammalian cell extracts, starting with the metabolites of the central carbon metabolism we regularly detected in our samples. This strategy is by no means limited to this kind of analysis and can be adapted to other platforms, other sample matrices and other metabolites of interest, e.g., plant secondary metabolites, lipids, drugs or steroid-hormones as long as standards are available. For this the compositions of the mixes should be adjusted to compounds of interest and capability of the analytical platform. In fact, we simply used the mixes optimized for GC–MS and measured these on a LC–MS metabolomics system. As in comparing the nominally similar columns from three different vendors we saw also in LC–MS non-linear shifts between experimental conditions.

These experiments clearly illustrate two features: First, with the help of these mixtures it is possible to transfer identifications between different setups or systems. Without prior knowledge regarding their retention behavior, a high number of compounds can be identified and a database can be created. Measuring and adding each compound individually would require dramatically more time.

Second, for both the GC–MS and LC–MS data, the retention differences between measurements of the same compound in different setups is similar or higher than the difference between compounds of the same class. Further non-linear shifts complicate the interpretation and the reliable identification of metabolites.

Preparation of these mixtures costs time and money: The metabolites have to be bought and stored, the composition has to be defined and improved, the mixtures need to be prepared and measured. Once the composition is defined, in 1–2 working days approximately 50 complete sets can be prepared that will last around 1 year when measured once a week. The cost for the compounds in the reported mixtures is approximately EUR 0.32 per set. The advantages in terms of data-reliability and reduced analysis time is worth these costs and efforts, even with more and also potentially more expensive compounds. We further recommend measuring the identification mixtures on each machine weekly as part of the quality control-routine or with each new project. In order to prevent human errors while working with different tables simultaneously, we highly recommend to code the identifications in the compound descriptions stored in the custom database, in human and machine-readable format as: Compound_Derivatisation-state_Retention_Ident-occurrence, e.g., “Alanine_(3TMS)_MP_RI:1367_IDENT:B+C”, with “MP” indicating the main product of the derivatisation in this case. This can include additional identifiers used for data storage or meta-analysis. For generating figures, the compound identification can be obtained by splitting the name after the second underscore, to ensure proper handling of different derivatisation products. The spectra-database with our 105 standards can be downloaded in NIST and .msp format from: https://github.com/KempaLab/Ident_Database.

## 4. Materials and Methods

### 4.1. Extracting Information from the KEGG Database

Compound information were extracted using the KEGG-API [11] and KEGGREST [20]. First the list of all KEGG-IDs was downloaded (on 25 March 2020), followed by the extraction of compound information (as name, sum-formula, weight, pathway). All inorganic metabolites, or metabolites without a formula and reaction as well as metabolites with a molecular weight above 1500 Dalton were removed. The human subset was created by downloading the list of human pathways and removing all metabolites that are not present in any of these pathways. Isomeric metabolites were counted based on molecular weight. For the low resolution dataset the weight was rounded to integers before counting.

### 4.2. Extracting Information from the HMDB Database

The “All Metabolites” dataset contained in the HMDB version 4.0, released on 16 January 2019, was used. We extracted the HMDB-ID, name, formula, average molecular weight, status, the KEGG ID, secondary accessions and names. Then we removed inorganic metabolites and metabolites with a weight above 1500 Da, resulting in 109,573 metabolites. Lipids were identified by text filter when the name contained two numbers separated by a colon, present in the majority of lipids indicating the length of the fatty acid and the number of double-bonds. For the “human” subset we only considered metabolites with status “detected” or “quantified”, resulting in 2976 metabolites. Isomeric metabolites were counted based on molecular weight, for the low resolution dataset the weight was rounded to integers before counting. Data was further aggregated using R [21].

### 4.3. Comparisons of Spectra and Retention Time Similarities in the GMD

The GMD was downloaded as msp-file from their webpage. Selected metabolites were compared pairwise using the compare feature in NISTMS-Search (AMDIS).

### 4.4. Preparation of the Ident-Mix

We started by compiling a list of metabolites regularly detected with our setup in biological samples. We consequently identified 105 metabolites whose retention indices should be validated routinely. We prepared stocks of each individual compound with a concentration of 1–10 mg/mL in 20% methanol or water, depending on solubility. We mixed these stocks according to Table S7 with amounts between 1 and 8 μL per sample. The amount was chosen based on MS-response, to give a clear peak with a reasonable intense signal. Consequently for 50 preparations 50 to 400 μL were aliquoted. To achieve similar compound amounts (0.5–20 μg per final aliquot) 82–100 μL were aliquoted 50 times for each of the four final Ident-Mix-compositions (A–D). The aliquots were dried in a rotational vacuum concentrator (Martin Christ, Osterode am Harz, Germany) without heating and dried stocks were stored at −20 ${}^{\circ }C$ until usage. Suppliers mark the compounds used as stable under these conditions and we did not observe a dramatic decrease in intensity during storage.

### 4.5. GC–MS Measurements

#### 4.5.1. Derivatisation for GC–MS

For derivatization, extracts were thawed in a rotational vacuum concentrator (Martin Christ, Osterode am Harz, Germany) without heating for 20 min. 20 μL of methoxyamine hydrochloride (40 mg/mL in pyridine) were added and samples were incubated for 90 min at 30 ${}^{\circ }C$. Next 80 μL of MSTFA containing 200 μg/mL *n*-alkanes (C_10_, C_12_, C_15_, C_17_, C_19_, C_22_, C_28_, C_32_, C_36_) as retention index markers were added and samples were incubated for 90 min at 37 ${}^{\circ }C$.

#### 4.5.2. Setup for GC–MS

Gas chromatography-mass spectrometry was carried out using a previously published method [22]. Gas chromatographic separation of metabolites was performed on an Agilent 6890 N (Agilent, Santa Clara, CA, USA) equipped with an Agilent VF-5ms column and coupled to a Pegasus III-TOF-MS-system (LECO Corp., St. Joseph, MI, USA). The initial temperature was held at 67.5 ${}^{\circ }C$ for 2 min, followed by a temperature gradient of 5 ${}^{\circ }C$/min until 120 ${}^{\circ }C$, then 7 ${}^{\circ }C$/min until 200 ${}^{\circ }C$, followed by 12 ${}^{\circ }C$/min until 320 ${}^{\circ }C$ with a hold time of 6 min. The transfer line was kept at 250 ${}^{\circ }C$ throughout. A cold injection system was used with a matching baffled deactivated liner (CIS4, Gerstel, Mülheim an der Ruhr, Germany), operating in split mode (split 1:5, injection volume 1 μL), with the following temperature gradient applied: hold of the initial temperature of 80 ${}^{\circ }C$ for 0.25 min, followed by a temperature increase of 12 ${}^{\circ }C$/s to 120 ${}^{\circ }C$, followed by a temperature increase of 7 ${}^{\circ }C$/s to 300 ${}^{\circ }C$ with a hold time of 2 min. Spectra were recorded in a mass range of 60–600u with 20 spectra/s, ionization energy (EI) was set to −70 eV, the ion source temperature was set to 250 ${}^{\circ }C$.

The setup is nearly identical to the setup used to generate the GMD, with some small exceptions: The helium flow was increased from 0.6 mL/min to 1.2 mL/min, the start temperature was decreased from 70 ${}^{\circ }C$ to 67.5 ${}^{\circ }C$, with a longer hold, and the main ramp was expanded from a single 9 ${}^{\circ }C$/min gradient to three gradients with 5, 7 and 12 ${}^{\circ }C$/min. The final temperature was decreased to 320 ${}^{\circ }C$. (see also Table S10) Furthermore, C_18_ retention index marker was replaced with a C_17_ retention index marker, as the retention index area of 1800 contained more important coeluting compounds.

#### 4.5.3. Peak Picking and Compound Annotation

Data was smoothed, deconvoluted and baseline corrected using ChromaTOF (LECO Corp., St. Joseph, MI, USA). Peaks were picked using ChromaTOF with a signal to noise threshold of 20. Compounds were initially identified using GMD spectra and later against a custom spectra database, build up gradually with spectra from the Ident-Mixes or compounds measured individually.

Initial annotation was performed by single measurements, or combinations of separately eluting compounds showing distinct mass spectra. To compare ther retention behaviour on three different columns (see below), we used the Ident-Mix as described in this manuscript.

#### 4.5.4. Column Type Comparison in GC–MS

To test the effect of different GC-columns we measured the Ident-Mixes on the same machine, equipped with three different columns from different vendors. All columns had identical properties with 5% diphenyl, 95% dimethyl polysiloxane coating material, a length of 30 m, inner diameter of 0.25 mm and film thickness of 0.25 μm. Tested columns were: TG-5SilMS (Thermo Fisher Scientific, Waltham, MA, USA), Rxi-5ms (Restek, Bad Homburg, Germany) and VF-5ms ((Agilent, Santa Clara, CA, USA)). On the three columns tested, we measured the Ident-Mixes and used Maui-VIA [23] to identify the substances. Even with considerable retention index shifts, with the help of the Ident-Mix it was still possible to identify the compounds in ca. 1 h per column. In a second run, using newly established RIs, the identifications/verifications took ca. 10–15 min.

### 4.6. LC–MS Measurements and Analysis

LC–MS measurements were performed at the metabolomics facility of the Beatson Institute for Cancer Research, Glasgow, UK. For this freeze dried samples were resuspended in 200 μL extraction solvent (methanol:acetonitrile: water, 5:3:2) shaken on ice and centrifuged ( 10 min at maximum speed and 4 ${}^{\circ }C$). The supernatant was transferred to LC–MS vials and stored at −80 ${}^{\circ }C$ until analysis. LC–MS measurements were performed as described elsewhere [24], utilizing high-resolution liquid chromatography (Thermo Fisher Scientific, Waltham, MA, USA) with a ZIC-pHILIC column (VWR) at pH 9.4 coupled via electrospray ionization (ESI) to an Orbitrap Q Exactive mass spectrometer (Thermo Fisher Scientific, Waltham, MA, USA). The same preparation was measured twice by LC–MS 11 months apart on the same machine with LC-column replaced in between. Raw data was processed using Compound Discoverer version 3.2 (Thermo Fisher Scientific, Waltham, MA, USA), following an unknown compound identification strategy. For this, retention times were aligned across data files (maximum shift 2 min, mass tolerance 5 ppm). Compound detection (minimum peak intensity 1,000,000) and grouping of compound adducts was carried out across all samples (mass tolerance 5 ppm, RT tolerance 0.2 min). Missing values were filled using the “Fill Gap” feature (mass tolerance 5 ppm, S/N threshold 10). In the first round ChemSpider node was used to suggest possible peak identifications (search by mass or predicted formula, mass tolerance of 5 ppm, databases: HMDB, KEGG and BioCyc). Suggested identifications were checked with the pattern in the Ident-Mix occurrence. Typically the most intense peak that matched to the pattern was used. Identifications of chemically similar compounds (e.g., mannose/glucose) were swapped towards the compound with the matching pattern. For all identified compounds a library was created as Masslist, containing the name, the pattern and the measured retention time. In the second round, identification was performed with the created Masslist, for the two different measurements (mass tolerance: 5 ppm, RT tolerance: 1 min). Except for one metabolite all metabolites contained in the Masslist were detected in both experiments. The results were finally compared with the retention times stored in an early version of the Beatson in-house database, derived from measuring a different set of authentic standards (personal communication).

## 5. Conclusions and Outlook

In this paper we present a method to measure a set of standards distributed over four different samples in parallel to the samples in a mass-spectrometry workflow. With the help of these mixtures it is possible to identify the compounds contained in the mixes with high confidence. The retention information can be updated for each experiment allowing smaller tolerance for the retention time shifts. As a consequence compounds with small differences in the database (as isomeric compounds) can still reliably identified. Strictly speaking the high level of confidence can only be guaranteed for compounds contained in the mixture. However, as this is a well defined set of many compounds over different compound classes it could be used to deduce the expected retention under changed conditions for other compounds, too. Further work is needed to test and evaluate the usability and limits of this strategy.

These mixtures represent a well-defined compound matrix that (in the best case) resembles the composition of the samples to be analysed, qualitatively and quantitatively. Therefore it is perfectly suited to set-up, test and improve the analytical methods. It can be used to test different analytical platforms (LC–MS or GC–MS), column types, temperature- or solvent-gradients and then compare results by identifying the number of metabolites with improved or worse separation between different strategies. Regarding data processing the peak detection parameters can be adjusted with this smaller subset to separate all important metabolites before applying these parameters to the full dataset containing the samples.

The Ident-Mix further generates a clear binary information about the presence of a compound. As the positive signal is always strong enough, the threshold for not-founds can be set quite high, so false-positive readings can be excluded. In the future this “binary” strategy could enable computer-algorithms to automatically identify all included metabolites, by combining the retention information and the identification based on *m*/*z* or mass-spectra with the occurrence matrix.

## Figures and Tables

**Table 1:** Identification parameters for common setups in metabolomics research.

Processing Step	GC-EI–ToF-MS	LC–Orbitrap-MS
Derivatisation	essential to mask polar groups	not essential but can be used selectively to target specific compound classes
Chromatographic resolution	narrower peaks (ca. 4–5 s)	wider peaks (ca. 20–30 s)
MS-resolution	typically unit-resolution	high resolution
Chromatographic identification	typically by retention index system	typically reliant on retention time
Compound identification	based on fragmentation	based on (exact) mass
Potential misident- ifications	metabolites with chemical similarity	metabolites with identical sum formula

**Table 2:** Composition of the Ident-Mix (excerpt from Table S7). Shown are three different metabolite classes with potential identification problems based on compound and RI similarity. Most metabolites generate two peaks due to multiple derivatisation products, often with the second peak lower abundant. However, for fructose both peaks have nearly the same intensity (Figure 4a,b), so we list the RI for both peaks here. Colors are given to illustrate appropriately the same colour scheme as used in Figure 4.

Class	Compound	Expected RI	Ident Mix Occurrence			
Pentoses	Xylose	1651	A		C	
	Ribose	1672		B		D
	Arabitol	1711			C	D
	Ribitol	1716	A	B		
Hexoses	Fructose	1862 and 1872		B	C	
	Mannose	1876	A			D
	Galactose	1880	A		C	
	Glucose	1886		B		D
Sugar Derivatives	Sorbitol	1926	A			D
	Glucuronic acid	1927	A		C	
	Glucosamine	1932	A	B		
	Galacturonic acid	1935		B		D
	Gluconic acid	1996		B	C	
			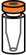	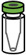	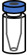	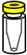

## Supplementary Materials

The following are available at https://www.mdpi.com/2218-1989/10/11/457/s1, Figure S1: Mapping of the Compounds Included in the Ident-Mix on KEGG-Map, #01100 Black dots represent a compound included in the Ident-Mix as reported in this publication, Table S1: Comparison of compound and isomer counts in KEGG and HMDB, Table S2: Isomeric distribution in KEGG, Table S3: Isomeric distribution in HMDB, Table S4: Isomeric compounds in KEGG, Table S5: Isomeric compounds in HMDB, Table S6: RI differences sugars and sugar, Table S7: Ident-Mix composition, Table S8: RI shifts between column vendors, Table S9: RT shifts between repeated measurements with different columns, same make and model, 11 months apart, Table S10: Temperature gradient comparison.

## Abbreviations

The following abbreviations are used in this manuscript:

CE–MS	Capillary electrophoresis mass-spectrometry
Da	Dalton
GC–MS	Gas chromatography coupled to mass-spectrometry
GMD	Golm Metabolome Database
HMDB	Human Metabolome Database
KEGG	Kyoto Encyclopedia of Genes and Genomes
LC–MS	Liquid chromatography coupled to mass spectrometry
MeOX	Methoxyamine
MSTFA	N-Methyl-N-(trimethylsilyl) trifluoroacetamide
*m*/*z*	Mass to charge ratio
RI	Retention index
