# Supplementary figures and images for: Towards a More Reliable Identification of Isomeric Metabolites Using Pattern Guided Retention Validation
Source: Metabolites. 2020 Nov 12;10(11):457. doi: 10.3390/metabo10110457 (PMC7696895; doi:10.3390/metabo10110457)

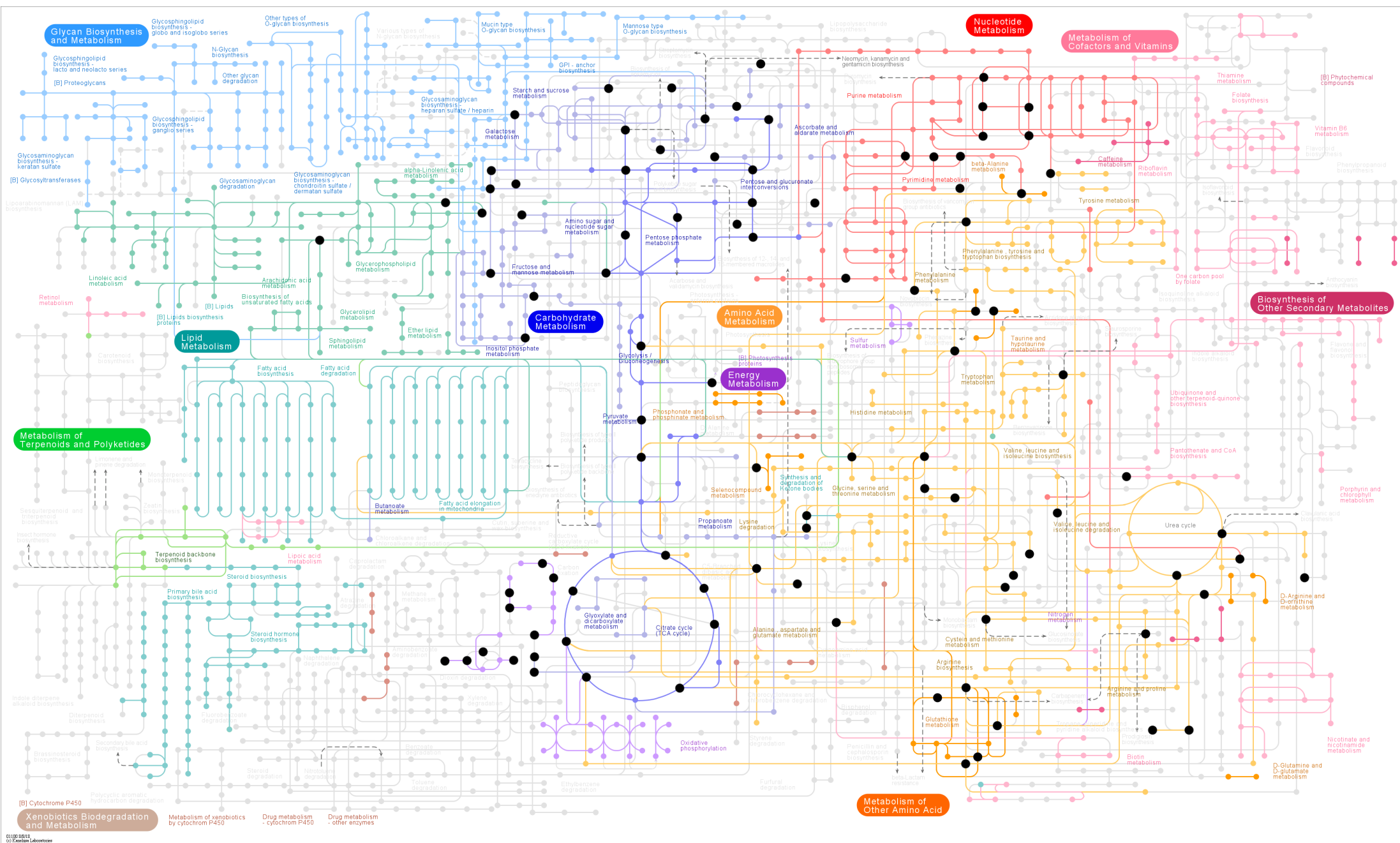

Supplement: Supplementary file 1 [file metabolites-10-00457-s001.zip › Supple/Supplementary_Figure_A1.pdf]
